# Modulation of *in vitro* Network Activity by Optogenetic Stimulation of Parvalbumin-positive Interneurons During Estrous Cycle

**DOI:** 10.2174/011570159X326861241129093354

**Published:** 2025-01-22

**Authors:** Fei Ran Li, Maxime Lévesque, Siyan Wang, Mia Gemayel, Massimo Avoli

**Affiliations:** 1 Departments of Neurology & Neurosurgery, and Physiology, Montreal Neurological Institute-Hospital, McGill University, 3801 University Street, Montréal, Québec, H3A 2B4, Canada;; 2 Department of Psychology, McGill University, 2001 McGill College, Montreal, Quebec, H3A 1G1, Canada

**Keywords:** Catamenial epilepsy, estrous cycles, parvalbumin-positive interneurons, optogenetic stimulation, 4-aminopyridine, progesterone blood levels

## Abstract

**Background:**

Catamenial epilepsy, which is defined as a periodicity of seizure exacerbation occurring during the menstrual cycle, has been reported in up to 70% of epileptic women. These seizures are often non-responsive to medication and our understanding of the relation between menstrual cycle and seizure generation (*i.e*. ictogenesis) remains limited.

**Methods:**

Here, we employed the *in vitro* 4-aminopyridine model of epileptiform synchronization, to analyze the effects induced by optogenetic activation of parvalbumin (PV)-positive interneurons at 8 Hz during estrous and non-estrous phases in female PV-ChR2 mice.

**Results:**

We found that: (i) optogenetic stimulation of PV-positive interneurons induced an initial interictal spike followed by field oscillations occurring more often in estrous (59%) than in non-estrous slices (17%); (ii) these oscillations showed significantly higher power in estrous compared to non-estrous slices (*p <* 0.001); (iii) significantly higher rates of interictal spikes and ictal discharges were identified in both estrous and non-estrous slices during optogenetic stimulation of PV-positive interneurons compared to periods of no stimulation (*p <* 0.05); and (iv) ictal events appeared to occur more frequently during optogenetic stimulation in estrous compared to non-estrous slices.

**Conclusion:**

Our findings show that optogenetic activation of PV-interneurons leads to more powerful network oscillations and more frequent ictal discharges in estrous than in non-estrous slices. We conclude that during the rodent estrous cycle, PV-interneuron hyperexcitability may play a role in epileptiform synchronization and thus in catamenial seizures.

## 1 INTRODUCTION

The menstrual cycle is associated with changes in estrogen and progesterone blood levels that can profoundly influence physiological brain excitability and thus behavior [1]. These sex hormonal changes can also play a role in neurological disorders such as focal epilepsy. Accordingly, epileptic women can report increased seizure occurrence/severity during their menstrual cycle. This condition is termed catamenial epilepsy [2-4], affects up to 70% of epileptic women [3, 5, 6], and is observed in focal epileptic disorders such as temporal lobe epilepsy [3, 7]. Catamenial seizures are often unresponsive to medications [6] and the fundamental mechanisms causing ovarian cycle-dependent changes in seizure severity remain unclear thus limiting the identification of effective pharmacological treatments.

In rodents, the menstrual cycle is termed estrous, and it is shorter than in humans since it lasts 4-5 days. The estrous cycle is divided into four phases that are identified as proestrus, estrus, metestrus, and diestrus [8]. The proestrus and estrus are characterized by high blood estrogen levels while the metestrus and diestrus are associated with high blood progesterone levels [9], and a common view is that pro- and anti-epileptogenic effects are caused by estrogen and progesterone, respectively [2, 5, 10-12]. Accordingly, Li *et al.* [13] have reported in the kainic acid model of temporal lobe epilepsy that seizure burden increases during proestrus/estrus.

A few years ago, Clemens *et al.* [14] discovered that cortical, parvalbumin (PV)-positive, inhibitory interneurons in rodents become hyperexcitable during the estrous phase. Such an increase in PV-positive interneuron excitability - which is mediated by the estrogen receptor β [14] - may lead to enhanced GABA_A_ signaling thus favoring epileptiform synchronization (see for review Avoli *et al.* [15] and de Curtis and Avoli [16]). Indeed, the sustained firing of interneurons has been reported at the onset as well as during focal seizures in several *in vivo* and *in vitro* studies performed in epileptic patients and in animal models [17-20]. Moreover, interneurons firing has been shown to be involved in physiological rhythms such as theta (4-10 Hz) oscillations [21].

Optogenetics allows for the reversible excitation or silencing of neurons with millisecond time resolution using light-activated ion channels and pumps that are expressed in target cell populations of transgenic animals [22, 23]. Therefore, in this study we used field potential recordings to analyze the responses generated by entorhinal cortex (EC) networks in brain slices perfused with 4-aminopyridine (4AP)-containing medium during optogenetic activation of PV-positive interneurons at 8 Hz. These slices were obtained from female PV-ChR2 mice at different phases of the estrous cycle.

## 2 MATERIALS AND METHODS

### 2.1 Animals

All procedures performed in this study were designed according to the guidelines of the Canadian Council on Animal Care and approved by the McGill University Animal Care Committee. PV-ChR2 female mice (60- to 130-old) were obtained from crossbreeding PV-Cre [B6;129P2-pvalbtm1(cre)Arbr/J, The Jackson Laboratory; RRID: IMSR_JAX:008069] with Ai32 mice [R26-lox-stop-lox-ChR2(H134R)-EYFP, The Jackson Laboratory; RRID IMSR_JAX:012569]. All lines were maintained in-house. The study is reported in accordance with ARRIVE guidelines.

### 2.2 Identification of the Estrous Phase

**Fig. (1):**
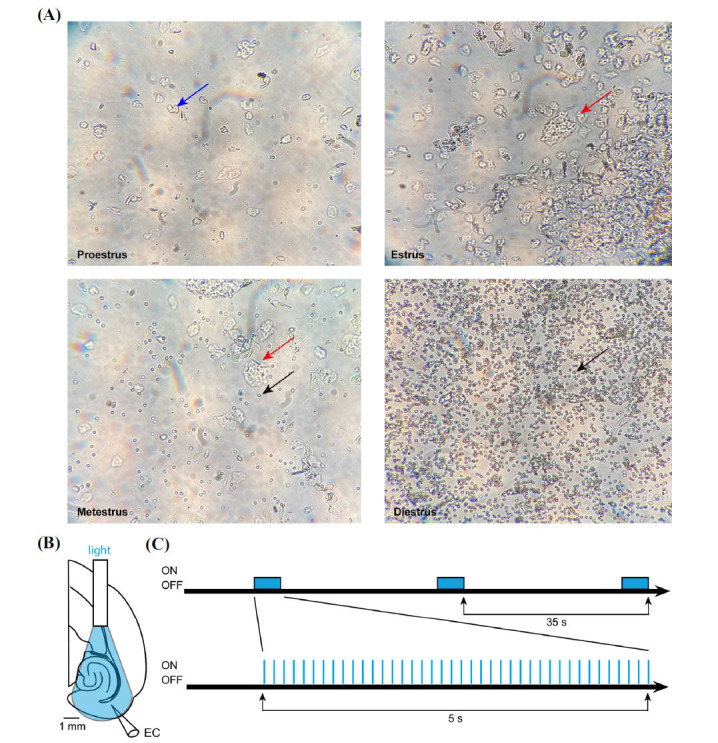
Estrous cycle identification and schematic diagram of the protocol. (**A**) Microscopic images showing the cell compositions of vaginal smears from proestrus, characterized by the presence of nucleated epithelial cells (blue arrow), estrus, characterized by the presence of cornified epithelial cells (red arrow), metestrus characterized by the presence of cornified epithelial cells (red arrow) and leucocytes (black arrow), and diestrus, characterized by the presence of leucocyte (black arrow). (**B**) Location of the recording electrode and the optic fiber. The shaded area indicates the estimated illuminated area (**C**) Diagram showing the optogenetic stimulation protocol (8 Hz optogenetic stimulation for 5 s followed by a 30 s period of no stimulation) after the application of 4-aminopyridine (4AP). EC, entorhinal cortex.

The estrous phase of the female mice used in our experiments was identified between 9:30 AM and 10:30 AM, 1 hour before they were decapitated, using the same procedures as in our previous study [24]. Samples from multiple females were collected at once. Animals in which we could not identify the phase of the estrous cycle were not included in this study. The phases of the estrous cycle were identified using vaginal smear samples (Fig. **1A**) [24, 25]. Stages of the estrous cycle were determined by observing the presence of leukocytes, cornified epithelial cells, and nucleated epithelial cells in the vaginal smear under a microscope (100x magnification). The proestrus and estrus were characterized by nucleated epithelial cells (blue arrow in Fig. **1A** - proestrus) and cornified epithelial cells (red arrow in Fig. **1A** - estrus) respectively, while the metestrus was characterized by the presence of cornified epithelial cells (red arrow in Fig. **1A** - metestrus) and leucocytes (black arrow in Fig. **1A** - metestrus). The diestrus was characterized by domination of leucocytes (black arrow in Fig. **1A** - diestrus). As proposed by Clemens *et al.* [14], the vaginal proestrous and estrous phases were combined into “estrus” since it is representative of the late follicular and very early luteal period of the human menstrual cycle, a time of increased seizure burden in women with periovulatory catamenial epilepsy [14, 26]. The vaginal metestrus and diestrus were defined as “non-estrus”.

### 2.3 Slice Preparation and Maintenance

As in our previous study [24], mice were deeply anesthetized with isoflurane and then transcardially perfused with ~25 ml choline cutting solution containing 132.5 mM choline chloride, 2.5 mM of KCl, 0.7 mM of CaCl_2_, 3 mM of MgCO_2_, 1.2 mM of NaH_2_PO_4_, 25 mM of NaHCO_3_, and 8 mM of glucose. They were then decapitated, and the brain was quickly removed and transferred into ice-cold, oxygenated (95% O_2_ and 5% CO_2_) choline-cutting solution. The brain was separated into two hemispheres and sliced horizontally with a vibratome (VT1000S; Leica, Wetzlar, Germany); 400-μm thick slices were transferred to a dish where they were kept under room temperature in artificial cerebrospinal fluid (ACSF) containing 124 mM of NaCl, 2 mM of KCl, 26 mM of NaHCO_3_, 2 mM of CaCl_2_, 2 mM MgSO_4_, 1.25 mM of KH_2_PO_4_, and 10 mM of D-glucose (pH 7.4, 305 mosmol/kgH_2_O) and humidified gas (O_2_/CO_2_, 95%/5%). Slices were allowed to recover for 1 hour in a dish and were then transferred to an interface chamber with 150 µM 4AP in the ACSF at a flow rate of 1-1.5 ml/min and a temperature of 30°C.

### 2.4 Electrophysiological Recordings

Local field potentials from the EC were recorded for approximately 5 min before 4AP application and then for 120 min. Glass pipettes (1B150F-4; World Precision Instruments, Sarasota, FL; tip diameter <10 µM, resistance 5-10 MΩ) were filled with ACSF. Signals were sampled at 5000 Hz, amplified with a high impedance amplifier and digitized (Digidata 1322 A, Molecular Devices, Palo Alto, CA). pCLAMP software (Molecular Device) was used to record signals on a computer.

### 2.5 Optogenetic Stimulation

Blue light pulses (450 nm, 25 mW, 50 mA) from a laser diode fiber light source (Doric lenses, Quebec, Canada) were delivered onto the mouse brain slices (Fig. **1B**) ~0.5 cm above the EC region of the slice. Stimulation was performed at 8 Hz since it has been shown that it is the optimal frequency to activate PV-interneurons and drive theta field oscillations [21, 27]. The optogenetic stimulation protocol - which was adapted from what was used by Wang *et al.* [28] in male mice - is shown in Fig. (**1C**). One round of optogenetic stimulation included 5 s of stimulation “ON” time followed by 30 s during which no stimulation was performed “OFF”. During each 5 s stimulation “ON” time, 40 light pulses, each lasting 20 ms, were delivered at 8 Hz. Each brain slice went through 3 to 15 stimulation rounds. Optogenetic protocol started 1 hour after the 4AP application. In the following figures, “ON” refers to the 5 s of light stimulation whereas “OFF” refers to the following 30 s of no stimulation. “No opto” refers to field potential recordings during which no optogenetic stimulation was performed, *i.e*. during the “OFF” condition in an optogenetic cycle and outside of optogenetic stimulation. To identify significant increases or decreases in interictal spike occurrence during optogenetic stimulation, values were shuffled 1000 times, and peaks above 2 SD of the shuffled mean were considered as significant.

### 2.6 Data Analysis and Statistics

**Fig. (2):**
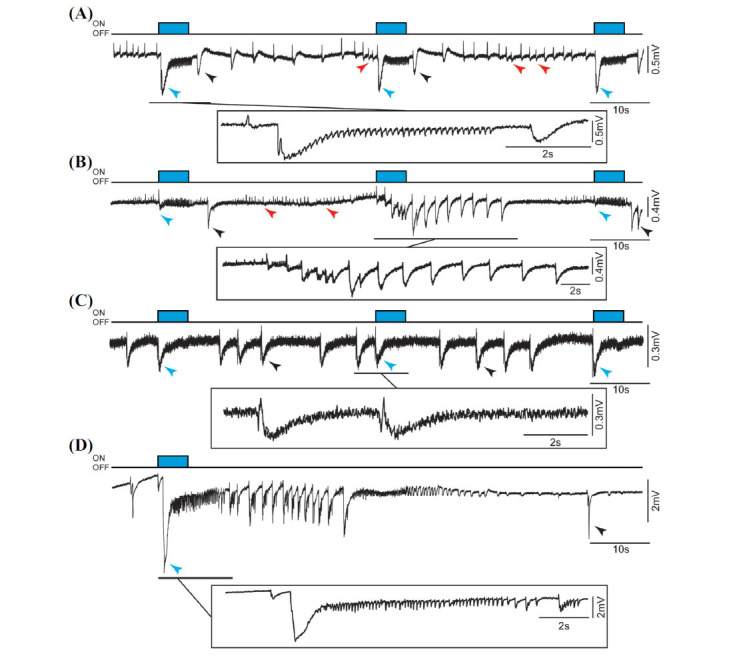
Field potential recordings obtained from the EC during application of 4AP and optogenetic stimulation of PV-positive interneurons at 8 Hz. Experiments shown in (**A**) and (**B**) were performed in brain slices obtained during estrus while recordings illustrated in (**C**) and (**D**) were obtained from brain slices during non-estrus. Note in panel A that a large negative-going interictal spike followed by 8 Hz field oscillations was consistently observed in this estrous slice while the same optogenetic procedure induced interictal events without any 8 Hz field oscillations in the non-estrous slice show in panel (**C**). Examples of the interictal spikes triggered by optogenetic stimulation are labelled with blue arrowheads, while black arrowheads indicate spontaneous interictal spikes under 4AP. Note also that, in a different estrous slice shown in panel B, this optogenetic stimulation protocol can evoke an ictal discharge. In a different non-estrous slice shown in panel (**D**), an ictal-like discharge was evoked by the last run of optogenetic stimulation. Insets in each panel illustrate specific portions of the field recordings on a shorter time scale. Small interictal spikes occurring at a frequency of approximately 1.5 Hz (red arrowheads) were not included in the analysis since they may represent continuous fast, CA3 driven interictal spikes originating from the CA3 region of the hippocampus.

In-house MATLAB (R2022b) scripts were used to analyze data. A 40 Hz Gaussian low-pass filter was applied to signals that were then down sampled to 1000 Hz. Artifacts in the signals were first removed through visual inspection. Ictal discharges, characterized as events longer than 5 s, were marked manually. Peaks with amplitude larger than 4SD of the mean of the average signal and lasting less than 5 s were identified as interictal spikes. Therefore, the small interictal spikes occurring at a frequency of approximately 1.5 Hz (Figs. **2A** and **B** red arrowheads) were not included in the analysis since they may represent continuous fast, CA3-driven interictal spikes originating from the CA3 region of the hippocampus [29]. The deflection from baseline was considered as the onset of interictal discharges and the end was marked as the return to baseline. The onset and end of each interictal discharge were automatically determined by considering the slope and area under the curve of each peak relative to the baseline neighboring signal.

Since data were not normally distributed, Wilcoxon Rank-Sum, Chi-square and Fisher exact probability tests were used. The level of significance was set at *p <* 0.05.

## 3 RESULTS

Fig. (**2**) illustrates field potential recordings during the application of 4AP and optogenetic stimulation of PV-interneurons at 8 Hz in brain slices that were obtained from transgenic mice during estrus (panels A and B) and non-estrus (panels C and D). Optogenetic stimuli induced an initial, predominantly negative-going interictal spike (blue arrowheads) that was consistently followed by 8 Hz field oscillations in estrous slices (Figs. **2A** and **B**) but less so in non-estrous slices (Figs. **2C** and **D**). Moreover, optogenetic stimulation could also evoke ictal discharges in both estrous (Fig. **2B** expanded) and non-estrous slices (Fig. **2D** expanded). To note that in both estrous and non-estrous slices we could also record spontaneous interictal spikes (black arrowheads) and spontaneous ictal-like events (not shown). In the following sections, we will analyze and compare in detail the field oscillations and epileptiform discharges triggered by optogenetic stimulation in estrous and non-estrous slices.

### 3.1 Field Oscillations

**Fig. (3):**
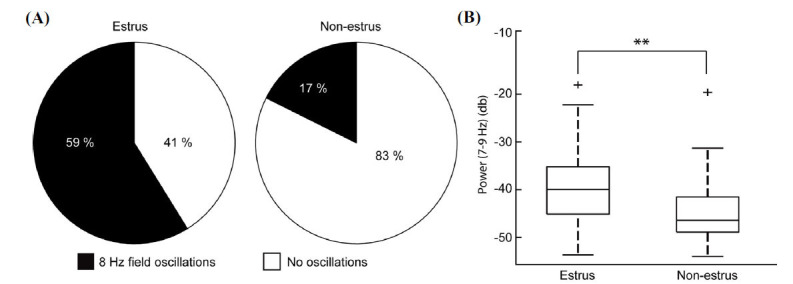
Application of 4-aminopyridine (4AP) and optogenetic stimulation-induced 8 Hz oscillations. (**A**) Proportion of slices showing 8Hz oscillations under the optogenetic stimulation protocol in estrous and non-estrous mice. (**B**) Bar graph showing the power of field oscillations. The oscillation power between 7 Hz and 9 Hz was quantified on a decibel scale. Slices from estrous mice (n=41 slices, 6 animals) had significantly higher power (** *p <* 0.001) compared to slices obtained from non-estrous mice (n=30 slices, 5 animals).

Quantification of the field oscillations that followed the initial interictal spikes induced by optogenetic activation of PV-positive interneurons at 8 Hz revealed that they occurred more often in estrus (59% of the trials, n=186/316 rounds of stimulations) than in non-estrus (17% of the trials, n=44/258 rounds of stimulations) (*p <* 0.001) (Fig. **3A**). Moreover, quantification of the oscillation power between 7 and 9 Hz indicated that slices obtained from estrous mice generated oscillations of higher power (-39.7 db) compared with those recorded from non-estrous (-44.61 db) mice (*p <* 0.001) (Fig. **3B**).

### 3.2 Interictal Spikes

**Fig. (4):**
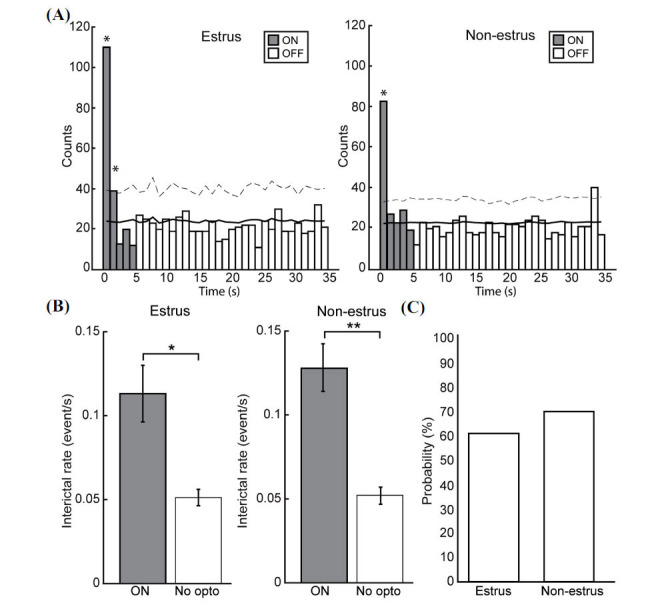
Temporal relation between interictal discharges and optogenetic stimulation. (**A**) Bar graphs showing the distribution of interictal discharges during the 35 s epoch (5 s of optogenetic stimulation (ON) and 30 s of no stimulation (OFF)). Note that interictal discharges mostly occur at the onset of the optogenetic stimulation protocol. The solid line shows the mean value after shuffling the histogram (1 000 times), whereas the dotted line shows the 2 SD of the shuffling procedure. (**B**) Rates of interictal discharges during the “ON” condition and when no optogenetic stimulation was performed (“No opto”). Interictal discharges occurred at significantly higher rates during the “ON” condition compared to the “No opto” condition in both estrous (n=41 slices, 6 animals, *p < 0.05) and non-estrous slices (n=29 slices, 5 animals, **p < 0.001). (**C**) Probability of optogenetic stimulations that triggered an interictal spike. No significant differences were observed. *p < 0.05.

Optogenetic stimulations at 8 Hz induced negative-going interictal spikes in both estrous and non-estrous slices (Fig. **2**, blue arrowheads). As illustrated in Fig. (**4A**), interictal spikes occurred throughout the optogenetic cycle but at higher rates during the “ON” condition compared to the “OFF” condition in both estrous and non-estrous slices (*p <* 0.05). When comparing rates of interictal spikes during optogenetic stimulation (“ON”) and when no stimulation was performed (“No opto”), their rates during the “ON” condition were significantly higher compared to the “No opto” condition for estrous mice (*p <* 0.05) and non-estrous mice (*p <* 0.001) (Fig. **4B**). When comparing the probability of triggering an interictal spike with optogenetic stimulation (“ON”) in estrous and non-estrous slices, similar values were observed (191 interictal spikes were triggered out of 315 runs of stimulation in estrous slices compared to 180 out of 258 runs in non-estrous slices) (Fig. **4C**).

### 3.3 Ictal Discharges

**Fig. (5):**
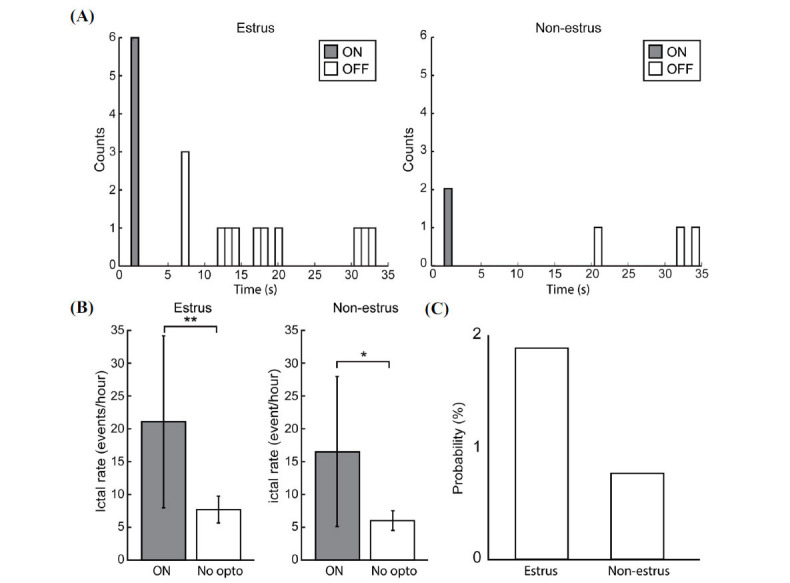
Temporal relation between ictal discharges and optogenetic stimulation. (**A**) Bar graph showing the distribution of ictal discharges during the 35 s epoch (5 s of optogenetic stimulation (ON) and 30 s of no stimulation (OFF)). Note that ictal discharges tended to occur at the onset of the optogenetic stimulation protocol. (**B**) Rates of ictal discharges during the “ON” condition and the “No opto” condition. In estrous slices, ictal rates were significantly higher in the “ON” compared to the “No opto” condition (n=41 slices, 6 animals *p* < 0.001). Similar results were observed in non-estrous slices (n=29 slices, 5 animals *p <* 0.05). (**C**) Probability of optogenetic stimulation triggering an ictal discharge. In estrous slices (6 ictal discharges triggered out of 315 runs of stimulation, 1.9%); this value was higher than in non-estrous slices (2 ictal discharges triggered out of 258 runs of stimulation, 0.77%). *p < 0.05, **p < 0.001.

Optogenetic stimulation at 8 Hz also triggered ictal discharges in both estrous and non-estrous slices (Fig. **5A**). Rates of ictal discharges during the 5 s optogenetic stimulation period (“ON”) were significantly higher than under the “No opto” condition in slices obtained from both estrous (*p <* 0.001) and non-estrous mice (*p <* 0.05) (Fig. **5B**). When comparing the probability of optogenetic stimulation triggering an ictal discharge in estrous (6 ictal discharges out of 315 rounds of optogenetic stimulation 1.9%) and non-estrous (2 ictal) discharges out of 258 rounds of optogenetic stimulation (0.78%) slices, we found that such probability was higher, although not significantly, in estrus compared to non-estrus (Fig. **5C**).

**Fig. (6):**
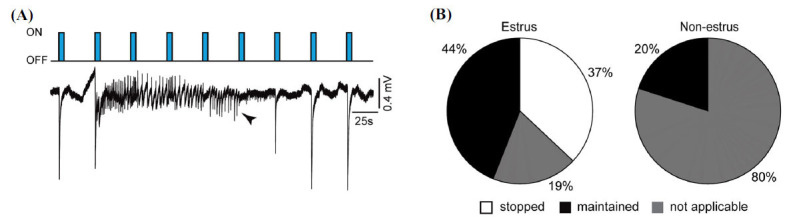
Optogenetic stimulation modulates ictal discharges. (**A**) Field recordings obtained in a brain slice from an estrous mouse showing an ictal discharge in response to optogenetic stimulation. Note that the ictal discharge persisted through the following rounds of stimulation but eventually stopped with the onset of the 5^th^ round (arrowhead). (**B**) Pie charts showing how ictal discharges were modulated with optogenetic stimulation. In estrous slices, ictal discharges could be maintained or stopped with optogenetic stimulation. In non-estrous slices, some ictal discharges were maintained under optogenetic stimulation. In both estrous and non-estrous slices, ictal events could also end before the next round of stimulation or they occurred following the last round (therefore termed “not applicable”). Note that such phenomenon occurred more often in non-estrous slices compared to estrous slices.

We also analyzed in estrous and non-estrous slices whether ictal discharges occurring during the “ON” condition were influenced by the following rounds of optogenetic stimulations. As shown in Fig. (**6A**), ictal activity triggered by 8 Hz stimulation of PV-positive interneurons could persists through the following rounds of optogenetic stimulation and eventually be stopped by a successive round (Fig. **6A**, arrowhead). We found in estrous slices that 44% of ictal discharges were maintained under optogenetic stimulation, whereas 19% of them were stopped; in 37% of cases, the ictal discharge ended before the next round of stimulation or was triggered by the last round of stimulation; these cases were therefore defined as “not applicable” (Fig. **6B**). In non-estrous slices, most ictal discharges (80%) stopped before the next round of optogenetic stimulation or were triggered by the last round of stimulation, whereas only 20% of them still persisted through consecutive rounds of stimulation (Fig. **6B**).

## 4 DISCUSSION

The main results of our findings can be summarized as follows: (i) optogenetic stimulation of PV-positive interneurons at 8 Hz triggered interictal spikes followed by field oscillations that occurred more often in estrous compared to non-estrous slices; (ii) these oscillations showed significantly higher power in estrous compared to non-estrous slices; (iii) significantly higher rates of interictal spikes and ictal discharges were observed in both estrous and non-estrous slices during optogenetic stimulation compared to when no optogenetic stimulation was performed; and (iv) ictal discharges appeared to occur more frequently during optogenetic stimulation in estrous compared to non-estrous slices.

### 4.1 The Estrous Phase Favors the Generation of Theta Oscillations

Previous studies have revealed that PV-positive interneurons play a major role in the generation of theta oscillations both in *in vitro* [21, 29, 30] and *in vivo* [31, 32] preparations. Furthermore, with the use of optogenetics, it was found that stimulation of PV-positive interneurons at 8 Hz is the optimal frequency to drive field theta oscillations in temporal lobe networks [21, 27] and that such oscillations could favor the generation of seizures in epileptic male mice [27] and in 4AP-treated brain slices obtained from male mice [28]. In this study, we now provide evidence that theta field oscillations driven by PV-positive interneuron stimulation at 8 Hz occur more frequently and have higher power in female estrous compared to non-estrous slices. High levels of estrogen during the estrous phase should contribute to the increased excitability of PV-positive interneurons [14] and thus to the generation of high-power theta oscillations. Presumably, such high excitability of PV-positive interneurons may also contribute to initiating and sustaining ictal activity.

### 4.2 Activation of PV-positive Interneurons Generates GABAergic-mediated Slow Interictal Spikes

Our results indicate that in both estrous and non-estrous slices - under 4AP treatment -, slow interictal spikes - presumably contributed by GABA_A_ signaling - are caused by the activation of PV-positive interneurons in the EC. Moreover, in the past two decades, it has been shown that, in the presence of 4AP, limbic networks can generate slow interictal spikes that are mostly due to postsynaptic activation of GABA_A_ receptors and are associated with transient increases in [K^+^]_o_ [33-35]. This conclusion is in line with previous findings obtained in male mice, in which optogenetic activation of PV-positive interneurons triggered GABA-mediated interictal spikes that persists under NMDA and non-NMDA receptor blockade [36]. Therefore, this type of interictal activity rests on the ability of PV-interneurons to synchronize populations of principal cells [37, 38] or the excessive activation of a large number of PV-positive interneurons that results in large-scale activation of GABA_A_ receptors [39].

### 4.3 High Levels of Estrogen could Facilitate Ictogenesis Through Increased Interneuron Excitability

Interestingly, ictal discharges generated from the activation of PV-positive interneurons occurred more frequently in estrous compared to non-estrous slices. However, this difference did not reach statistical significance, probably due to the small number of stimulations that were able to trigger ictal discharges out of a large number of trials. Nonetheless, our findings suggest that during the estrous phase, high blood levels of estrogen facilitate ictogenesis by increasing the excitability of interneurons [14]. Many studies have reported that interneurons increase their firing rates at seizure onset [18-20, 40-42] and recent evidence indicates that interneuron excitability *in vitro* is decreased with PHTPP, an estrogen receptor β-mediated blocker [14]. In line with these findings, kainic acid-treated female mice show a high seizure burden during the estrous phase [13].

## CONCLUSION

Our findings bring further evidence supporting the view that the estrous phase in rodents would favor neuronal excitability, the generation of field oscillations, and of epileptiform activity. Such increased epileptiform synchronization during the estrous phase may depend, at least in part, on the increased excitability of interneurons. We are inclined to propose that this pathological activity could underlie the generation of seizures in C2 catamenial epileptic women, during which seizures are more likely to occur around ovulation [43].

## Data Availability

All data generated or analyzed during this study are included in this published article.

## LIST OF ABBREVIATIONS

ACSF: Artificial Cerebrospinal Fluid
4AP: 4-aminopyridine
EC: Entorhinal Cortex
PV: Parvalbumin
